# Effectiveness of Digital Behavioral Activation Interventions for Depression and Anxiety: Systematic Review and Meta-Analysis

**DOI:** 10.2196/68054

**Published:** 2025-06-17

**Authors:** Eric Jia, Jushawn Macon, Michelle Doering, Joanna Abraham

**Affiliations:** 1 Department of Computer Science & Engineering Washington University in St. Louis St. Louis, MO United States; 2 Division of Biology and Biomedical Sciences Washington University School of Medicine St. Louis, MO United States; 3 Bernard Becker Medical Library Washington University School of Medicine St. Louis, MO United States; 4 Department of Anesthesiology Institute for Informatics, Data Science and Biostatistics (I2DB) Washington University School of Medicine St. Louis, MO United States

**Keywords:** anxiety, behavior, behavior change, cognition, cognitive, cognitive behavioral therapy, databases, depression, digital behavioral activation, digital health, digital intervention, disability, effectiveness, eHealth, health care, internet-based behavioral activation, mental health, meta-analysis, psychotherapy, quality of life, remote health care, stress, systematic reviews, telehealth, therapy

## Abstract

**Background:**

As digital interventions gain prominence in mental health care, they present opportunities to improve access and scalability. Despite their potential, the overall impact of digital behavioral activation (BA) interventions across different formats and populations is not yet fully understood.

**Objective:**

This systematic review examines the characteristics and functions of digital BA interventions and evaluates their effectiveness for mental health and other patient-related outcomes.

**Methods:**

A comprehensive search of databases (PubMed, Embase, Web of Science, APA PsycInfo, and ClinicalTrials.gov) was performed in November 2023 to identify randomized controlled trials (RCTs) assessing the effectiveness of digital BA interventions for depression and anxiety. A total of 2 reviewers screened the studies for inclusion. Meta-analysis using a random-effects model assessed intervention impact on outcomes including depression, anxiety, quality of life (QoL), BA scores, functioning and disability, and stress. Statistical heterogeneity was evaluated with the *I*² statistic and statistical significance was evaluated with *P* values. Studies that did not meet the meta-analysis criteria underwent narrative synthesis.

**Results:**

A total of 18 articles reporting 17 RCTs were included across three intervention types: (1) internet-based BA (n=12, 71%), delivering digital therapies to foster new behavioral activities for depression management; (2) electronic messaging–based BA (n=2, 12%), involving prompts to support behavior change; and (3) telehealth-based BA (n=3, 17%), providing remote health care services. We identified single-component and multicomponent interventions that combined BA with elements such as problem-solving therapy or cognitive behavioral therapy. A total of 12 RCTs were included in the meta-analysis, while the remaining studies were narratively synthesized. Risk of bias (RoB) was assessed in all included studies. Digital BA interventions significantly reduced depressive symptoms at 2 months (*P*<.001, *I*²=0%), 3 months (*P*=.001, *I*²=51%), and 6 months (*P*=.009, *I*²=29%) post treatment, but not at 12 months (*P*=.82, *I*²=89%). Significant improvements in BA scores at 6 months were observed (*P*<.001, *I*²=0%). QoL improved significantly at 3 months (*P*=.002, *I*²=22%) and 6 months (*P*=.009, *I*²=0%). Stress levels were also significantly reduced at 3 months (*P*<.001, *I*²=25%). However, no significant changes were identified in anxiety and functioning and disability outcomes at either 3 months (anxiety: *P*=.08, *I*²=68%) or 6 months (anxiety: *P*=.24, *I*²=44%; functioning and disability: *P*=.88, *I*²=90%). Across included studies, RoB was generally low, particularly for random sequence generation and allocation concealment.

**Conclusions:**

Digital BA interventions are effective in reducing depressive symptoms and improving QoL in the short- to midterm. However, these effects tend to diminish over time with no sustained benefits observed at 12 months. Future research should focus on developing and testing interventions with greater long-term efficacy, clarifying the role of BA within multicomponent digital approaches, and identifying the optimal intervention “dose” needed to maintain lasting effects.

## Introduction

Depression and anxiety persist as prevalent and debilitating mental health threats worldwide [1-3] and significantly impact an individual’s quality of life (QoL) [4,5], functioning [6,7], and overall health outcomes [8,9]. Various psychotherapeutic treatments help alleviate symptoms of depression and anxiety, including psychosocial education, mindfulness, and cognitive behavioral therapy (CBT) [10]. CBT is a widely used and effective behavioral treatment shown to reduce depression symptoms and anxiety [11-13]. CBT is a structured, goal-oriented talk therapy that helps individuals recognize how their beliefs may influence their actions, unlearn negative thoughts, and replace them with healthier thinking patterns and habits. Behavioral activation (BA), a form of CBT, aims to improve individuals’ mood and functioning by encouraging them to engage in activities that align with their personal values [14]. BA has been found to be effective in reducing depression and anxiety severity in young adult populations and various cultural populations across the world [15,16].

Due to the growing use of and advancements in digital health technologies, BA has been adapted over the past decade into digital formats that offer accessible and scalable treatment options [17]. In addition, digital BA treatments delivered via web-based platforms and mobile apps address other social and structural barriers to accessing mental health services, such as mental health stigma [18], insufficient mental health resources, and costs associated with traditional in-person therapy [19].

A systematic review of 9 randomized controlled trials (RCT) highlighted the efficacy of internet-based BA (iBA) interventions such as smartphone apps and websites for treating depression and anxiety symptoms and improving QoL [20]. Of these 9 interventions, 6 solely used BA, and 3 combined BA with other therapy components like problem-solving therapy and acceptance and commitment therapy (ACT). The review concluded that iBA interventions showed promise to be as effective as traditional face-to-face methods at reducing various forms of depression including subthreshold depression, postpartum depression, and depression with comorbid chronic conditions like diabetes. A similar updated review [21] assessing the cumulative efficacy of iBA interventions in treating depression symptoms and anxiety concluded that iBA interventions were more effective at reducing depression symptoms and anxiety severity immediately postintervention than control groups, which primarily included treatment as usual (TAU) and active controls such as digital psychoeducational materials and telephone support calls. However, the review also reported no significant improvement in depressive symptoms or anxiety at 6-month follow-up. Despite the invaluable insights on the nature and impact of iBA interventions on depression and anxiety, the scope of these reviews remains limited to only iBA interventions. Therefore, we currently lack a comprehensive understanding of the different types of digital BA interventions and their overall impacts in treating mental health conditions. To address this gap, we conducted a systematic review and meta-analysis to synthesize and appraise the empirical evidence on the effectiveness of various types of digital BA interventions including electronic messages, smartphone apps, and telehealth meetings for reducing either depression symptoms or anxiety, or both.

Our review objectives were 3-fold: first, to characterize the nature and functions of digital BA interventions; second, to ascertain the impact of digital BA interventions on patient outcomes; and finally, to highlight gaps in research and future directions for digital BA intervention research and its use in practice.

## Methods

### Search Strategy

The published literature was searched for RCTs on digital BA interventions for depression and anxiety using strategies created by a medical librarian (MD) and established using a combination of standardized terms and keywords. The search was run on November 15, 2023, without any date limits, in the databases Embase.com, Ovid MEDLINE, Web of Science, PubMed, Clinicaltrials.gov, and APA PsycInfo from database inception. The search was restricted using an English-language filter and the Cochrane-approved RCT filter was used in Embase, Ovid MEDLINE, and Web of Science. The search in APA PsycInfo was restricted using its methodology filter for clinical trials. Full search strategies are provided in Multimedia Appendix 1.

### Study Screening and Selection

Titles and abstracts of the retrieved articles were screened for eligibility by one reviewer (JM) using Covidence, a web-based collaboration software platform that streamlines the production of systematic reviews [22]. Eligible studies included RCTs of digital mental health interventions using BA in adults older than 18 years who screened positive for symptoms of either anxiety or depression, or both. Digital mental health interventions were defined as evidence-based digital interventions to prevent, manage, or treat mental disorders or diseases [23], including smartphone and tablet apps, internet-based programs, virtual reality, media-based programs, video games, computer programs, chatbots, telehealth, social media, podcasts, and webinars. Only original research articles published in peer-reviewed journals were included (refer to Multimedia Appendix 2 for inclusion criteria). The following types of articles were excluded: feasibility studies, study protocols, studies in pediatric patients, retrospective studies, design studies, evaluation studies of nondigital BA interventions, conference abstracts, and qualitative studies (Multimedia Appendix 3 for excluded studies).

Titles and abstracts that met the inclusion criteria and seemed relevant were retrieved for full-text review. A total of 2 reviewers (JM and EJ) independently assessed full-text articles for inclusion and disagreements were discussed and resolved with a third reviewer (JA). References from included articles were also screened for eligibility in line with standard practice to reduce the risk of publication bias and to identify as much relevant evidence as possible [24].

### Data Extraction and Management

A data abstraction form for extracting the relevant data from the included studies was iteratively developed and pilot-tested (refer to Multimedia Appendix 4 for the data extraction template). The final extraction was duplicated by 2 reviewers (JM and EJ), who independently extracted data pertaining to the population, intervention, comparison group, and outcomes (PICO) characteristics. Data discrepancies were reviewed and adjudicated by a third reviewer (JA).

### Data Analysis and Synthesis

A total of 2 reviewers (JM and EJ) coded the extracted data from the included studies. For example, to fully characterize the nuances underlying the interventions, the reviewers coded the technology type, intervention design approaches, theories driving the intervention, and so on (refer to Multimedia Appendix 5 for definitions of terms used in the review). All outcomes reported in the included studies were retrieved and organized as a matrix to avoid reporting bias. Studies reporting similar outcomes were pooled for meta-analysis and narrative synthesis.

### Meta-Analysis

We conducted a meta-analysis across studies to ascertain the effects of digital BA interventions on the selected outcomes. RCTs that reported similar outcomes (with ≥2 studies) were included in the meta-analysis. Standard mean differences with corresponding 95% CIs were evaluated in the meta-analysis since studies with similar outcomes often used different measures for either anxiety or depression, or both [25]. RCTs were excluded from a meta-analysis if they had insufficient data needed for a pooled analysis. However, where possible, missing aggregate data such as the aggregate SD for 2 separate trials within the same study were estimated through weighted averages using the provided means and SDs from each subgroup. A random-effects model was used, statistical heterogeneity was assessed using the *I*^2^ test statistic, and the level of significance was set at *P*≤.05. All analyses were conducted using RevMan (version 5; The Cochrane Collaboration).

### Narrative Synthesis

Narrative synthesis was conducted for studies corresponding to RCTs with insufficient reporting of outcome data, outcomes with a small number of studies (<2 studies per outcome), and studies that compared more than one intervention to the control; these studies had outcomes that could not be pooled for the meta-analysis or vote counting.

### Risk of Bias Assessment

The Cochrane risk of bias (RoB) 1.0 Tool [26] was used to assess the quality of RCTs as having high, low, or unclear bias across 7 different domains: random sequence generation, allocation concealment, blinding of personnel and participants, blinding of outcome assessment, incomplete outcome data, selective outcome reporting, and other bias. RoB was independently assessed by 2 reviewers (JM and EJ) and adjudicated by a third reviewer (JA). Approximately 10% of the data required further review due to disagreements resolved through team discussion until 100% consensus was reached.

## Results

**Figure 1:**
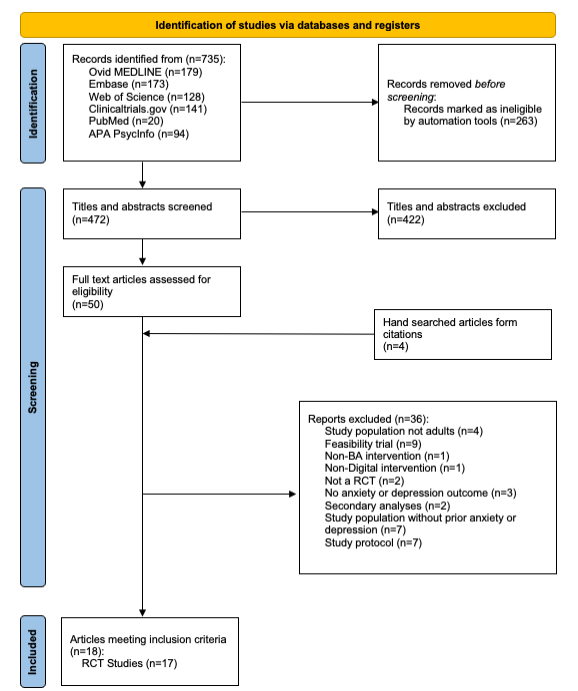
PRISMA study selection process [29]. BA: behavioral activation; RCT: randomized controlled trial.

Our search yielded a total of 735 citations which were imported into the Covidence system. After removing 263 duplicates identified by Covidence, 472 articles were selected for initial title and abstract screening. After title and abstract screening, 50 full-text articles were retrieved for full-text review of which 34 (68%) articles were excluded. As such, 4 additional articles were identified through manually screening reference lists of the included studies; 2 of the manually screened studies were excluded. Based on our full-text review, 18 articles met the eligibility criteria; however, 2 articles reported on the same intervention [27,28], resulting in a total of 18 included articles with 17 RCTs evaluating DBA interventions (Figure 1 [29]).

### Characteristics of Included Studies

The included 17 RCTs were published between 2013 and 2023 [27,28,30-45], with 6 RCTs (35%) published in 2023 [30,32,36,41,45]. A total of 7 RCTs (41%) were conducted in North and South America [27,28,30-35] and the remaining 10 RCTs (59%) in Europe. A total of 6 RCTs (35%) recruited participants from health care settings [27,28,32-36] and the rest from the general public through methods such as advertisements [30,31,37-41], health insurance companies [42,43], a corporate data warehouse [33], and regional associations of school psychologists [44]. Table 1 presents the characteristics of the included studies.

**Table 1:** Included study characteristics.

Author (year)	Country	Study arms	Sample size	Population	Recruitment setting	Intervention type	Control type
Araya et al (2021) [35]	Brazil and Peru	2	Intervention: 657 Control: 655	Adults receiving treatment for hypertension or diabetes with a PHQ-9^a^ score higher than 10	Health care	iBA^b^	EUC^c^
Birney et al (2016) [31]	United States	2	Intervention: 150 Control: 150	Employed adults with mild-to-moderate depressive symptoms (PHQ-9)	General public	iBA	Additional resources about depression
Buntrock et al (2015) [42]	Germany	2	Intervention: 202 Control: 204	Adults suffering from subthreshold depression (CES-D^d^)	General public	iBA	Non-BA^g^ digital intervention
Carlbring et al (2013) [37]	Sweden	2	Intervention: 40 Control: 40	Adults with a MADRS-S^e^ between 15-30 y	General public	iBA	WLC^f^
Choi et al (2020) [27] and Marti et al (2021) [28]	United States	3	BA^g^: 99 PST^h^: 98 Control: 98	Homebound adults > 50 y with moderate-to-severe depressive symptoms (HAMD^i^) in Central Texas	Health care	Telehealth	PST digital intervention, or Telephone support calls
Dahne et al (2023) [30]	United States	2	Intervention: 103 Control: 47	Adults who smoke >5 cigarettes per day and with symptoms of elevated depression (PHQ-8)	General public	iBA	TAU^j^
Danaher et al (2023) [32]	United States	2	Intervention: 96 Control: 95	Perinatal adult women with depression recruited from NorthShore University HealthSystem	Health care	iBA	TAU
Ebert et al (2014) [44]	Germany	2	Intervention: 75 Control: 75	Working teachers with depressive symptoms (CES-D)	General public	iBA	WLC
Guertler et al (2023) [36]	Germany	2	Intervention: 227 Control: 229	Adults who experienced subthreshold depression (*DSM-IV*^k^)	Health care	Electronic messages	TAU
Jelinek et al (2020) [38]	Germany	3	Intervention: 37 Control: 32 TAU: 35	Adults between 18-65 y with depressive symptoms	General public	iBA	TAU or active control
Ly et al (2013) [40]	Sweden	2	Intervention: 40 Control: 41	Adults with a score of at least 5 on the PHQ-9	General public	iBA	Non-BA digital intervention
Ly et al (2015) [39]	Sweden	2	Intervention: 46 Control: 47	Adults with depressive symptoms	General public	Hybrid (iBA + face to face sessions)	Nondigital BA intervention
Mueller-Weinitschke et al (2023) [41]	Germany	2	Intervention: 64 Control: 64	Adults between 18-65 y with depressive symptoms (PHQ-9 and QIDS-C^l^)	General public	iBA	TAU
Naik et al (2019) [33]	United States	2	Intervention: 136 Control: 89	Veterans with uncontrolled diabetes and depressive symptoms in Southeast Texas	Health care	Telehealth	EUC
Nobis et al (2015) [43]	Germany	2	Intervention: 129 Control: 127	Adults with diabetes and comorbid depressive symptoms (CES-D)	General public	iBA	Non-BA digital intervention
Sanabria-Mazo et al (2023) [45]	Spain	3	BA: 78 ACT^m^: 78 Control: 78	Adults between 18-70 y with a diagnosis of chronic lower back pain and displaying moderate-to-severe depressive symptoms (PHQ-9)	Health care	Telehealth	ACT digital intervention, or TAU
Scazufca et al (2024) [34]	Brazil	2	Intervention: 298 Control: 305	Adults>60 y with depressive symptoms (PHQ-9)	Health care	Electronic messages	EUC

^a^PHQ-9: Patient Health Questionnaire-9.

^b^iBA: internet-based behavioral activation.

^c^EUC: enhanced usual care.

^d^CES-D: Center for Epidemiologic Studies Depression Scale.

^e^MADRS-S: Montgomery–Åsberg Depression Rating Scale.

^f^WLC: Wait List Control.

^g^BA: behavioral activation.

^h^PST: problem-solving therapy.

^i^HAMD: Hamilton Depression Rating Scale.

^j^TAU: treatment as usual.

^k^DSM-IV: Diagnostic and Statistical Manual of Mental Disorders (Fourth Edition).

^l^QIDS-C: Quick Inventory of Depressive Symptomatology - Clinician Rated.

^m^ACT: acceptance and commitment therapy.

### Population

By design, all studies targeted populations with depression. A total of 2 RCTs (12%) had an inclusion criterion of age being greater than 50 years [27,28,34], while 3 other RCTs (24%) had an age cap [38,41,45]. A total of 2 RCTs with age caps limited participation to adults 18-65 years of age [38,41], while the third RCT was limited to 18-70 years [45]. The remaining 12 RCTs (70%) included all patients older than 18 years of age.

However, these RCTs had varying participant inclusion criteria: 3 RCTs (18%) enrolled patients directly from health care settings [34-36], 5 (28%) enrolled patients with access to the internet [31,32,38,42,44], 3 (18%) involved patients living in Sweden [37,39,40], 2 (12%) enrolled patients who could understand Spanish [27,28,45], 1 (6%) enrolled patients who were heavy smokers [30], 2 (12%) involved patients with German health insurance [41,43], and 1 (6%) focused only on veteran populations in Texas [33].

### Interventions

A total of three major types of digital BA interventions were identified across studies: (1) iBA interventions that deliver web-based therapies through self-guided or minimally guided digital platforms that help patients with depression develop new behavioral activities; (2) electronic messaging-based BA interventions that use messaging technologies to deliver periodic prompts and support via SMS, email, or app notifications to encourage behavior change: and (3) telehealth-based BA interventions that offer real-time, clinician-led therapy through remote communication tools like videoconferencing or telephone calls, aiming to provide BA services remotely. With 17 interventions total (due to 2 studies analyzing the same intervention), 12 interventions (71%) used iBA, encompassing interactive websites or smartphone apps [30-32,35,37-44], including all 6 self-paced interventions [30,31,37,38,40,42]. A total of 2 (12%) interventions used electronic messaging-based BA, involving SMS or emails, [34,36] and the remaining 3 (17%) interventions used telehealth to deliver BA over the phone [27,28,33,45].

### Intervention Components

A total of 9 (52%) RCTs reported interventions that used single-component digital BA interventions [27,28,30,34-36,38-40,45]. The remaining RCTs used multicomponent BA interventions. A total of 3 (18%) interventions incorporated problem-solving therapy in addition to BA [42-44]. A total of 2 (12%) interventions also included CBT [31,32] and one of these incorporated mindfulness in addition to CBT and BA [31]. One (6%) intervention used ACT with BA [37]. One (6%) intervention added the Health Action Process Approach (HAPA) [41]. Finally, one (6%) intervention used BA in addition to the 5 A’s Model (Assess, Advise, Agree, Assist, and Arrange) to Coping with Chronic Illness (Table 2) [33].

**Table 2:** Intervention characteristics. Sessions consist of structured lessons or modules that guide users through a predefined learning path.

Author (year)	Name	Technology	Components	Mechanisms of change	Dose: length	Dose: frequency	Guidance	Available support
Araya et al (2021) [35]	N/A^a^	Smartphone app	BA^b^	Participating in meaningful activities	6 wk	3 mini sessions (<10 min) per week	Guided	Initial face-to-face meeting, app preinstalled on device, nurse assistants provided support and called patients twice at beginning, assistants called when system detected nonadherence, patients could request tech support
Birney et al (2016) [31]	Moodhacker	Smartphone app	BA, CBT^c^, mindfulness	Added daily structure and reinforcement	6 wk	Daily mood and activity tracking	Self-guided	Daily scheduled emails providing guidance, tips, and reminders
Buntrock et al (2015) [42]	GET.ON	Web based	BA, PST^d^	Participating in meaningful activities, problem-solving	3-6 wk	One or two 30-min sessions per week	Guided	Guidance provided by web-based trainers focus on supporting participants to work through the exercises. Participants communicate with their trainer trough the internal messaging function of the system on which the intervention is implemented
Carlbring et al (2013) [37]	Depressionshjälpen	Web based	BA, ACT^e^	Participating in meaningful activities, diffusions, and values	7 wk	1 session per week	Self-guided	Internet-therapist assisted with administration, reading and sending feedback to the participant
Choi et al (2020) [27] and Marti et al (2021) [28]	N/A	Telehealth	BA	Participating in meaningful activities	5 wk each	1 session per week in each study	Guided	Telehealth
Dahne et al (2023) [30]	Goal2Quit	Smartphone app	BA	Participating in meaningful activities	8 wk	Regular activity tracking	Unguided	Help downloading app and a brief overview on the app
Danaher et al (2023) [32]	MomMoodBooster	Web based	BA, CBT	Participating in meaningful activities, social support, mood tracking	12 wk	1 session every 2 wk	Guided	2 scheduled calls for resolving difficulties and feedback
Ebert et al (2014) [44]	Alles onder controle	Web-based	BA, PST	Problem solving, coping skills, identifying important values	7 wk	5 total sessions	Guided	Feedback on modules completed
Guertler et al (2023) [36]	Actilife	Electronic messages	BA	Participating in meaningful activities, positive thinking, exercise, stress management, encouraging seeking help when needed	6 mo	Max of 3 feedback letters and 24 feedback messages	Guided	Not specified
Jelinek et al (2020) [38]	N/A	Web-based	BA	Mood tracking, participating in meaningful activities, problem solving	4 wk	Daily planned activities and worksheets	Unguided	Provided in program
Ly et al (2013) [40]	N/A	Smartphone app	BA	Daily structure, encourage social activities, participating in meaningful activities	8 wk	Daily behavior tracking	Self-guided	Brief therapist contact
Ly et al (2015) [39]	N/A	Hybrid (smartphone app and face-to-face)	BA	Positive thinking, positive reinforcement	9 wk	4 total face-to-face sessions and daily behavior tracking	Guided	4 in-person BA sessions
Mueller-Weinitschke et al (2023) [41]	InterAKTIV	Web based	BA, HAPA^f^	Participating in meaningful activities, problem solving	8 wk	7 total 45-min sessions	Guided	Written semistandardized feedback by an e-coach
Naik et al (2019) [33]	Healthy Outcomes Through Patient Empowerment	Telehealth	BA, Goal setting	Participating in meaningful activities, improving wellness, diet, physical activity, medication management, relaxation, problem-solving	6 mo	Biweekly during months 1-3 and monthly during months 4-6	Guided	Active coaching
Nobis et al (2015) [43]	GET.ON Mood Enhancer Diabetes	Smartphone app	BA, PST	BA, problem-solving, diabetes management, and concerns	6 wk	1 45-min session per week	Guided	Assistance offered through phone call and email if no activity within 7 days
Sanabria-Mazo et al (2023) [45]	N/A	Telehealth	BA	Participating in meaningful activities	8 wk	One 90-min session per week	Guided	Group therapy
Scazufca et al (2024) [34]	Vida semi standardized	Electronic messages	BA	Participating in meaningful activities, coping strategies	6 wk	2 messages a day for 4 d a week	Guided	Tech support contact

^a^N/A: not applicable

^b^BA: behavioral activation.

^c^CBT: cognitive behavioral therapy.

^d^PST: problem-solving therapy.

^e^ACT: acceptance and commitment therapy.

^f^HAPA: Health Action Process Approach.

### Intervention Delivery

Only 6 of the 17 (35%) digital BA interventions were self-paced, where participants could progress through the intervention at their own pace [30,31,37,38,40,42]. With the exception of 2 (12%) unguided [30,38] and 3 (18%) self-guided interventions [31,37,40], the remaining 12 (71%) interventions were guided, meaning that they contained predetermined topics and materials based on a standard protocol and were delivered by trained nurses or research assistants [27,28,32-36,39,41-45].

Only 1 (6%) intervention was delivered in a hybrid manner, with a smartphone app and in-person sessions delivered by MSc Clinical Psychologist Program final-year students who had completed their clinical training [39]. The interventions had a mean (SD) duration of approximately 8.1 (SD 7.3) weeks across all studies (N=17).

A total of 14 RCTs (82%) used interventions with shorter dosage lengths of 3-6, [42] 4, [38] 5, [27,28] 6, [31,34,35,43] 7, [37] 8, [30,40,41,45], and 9 weeks [39]. The remaining interventions had longer dosage lengths of 12 weeks [32] and 6 months [33,36].

The included study interventions were delivered either by sessions (eg, lessons or modules) [27,28,32,33,35,37,41-45] or interactions (eg, activity scheduling, messages, or behavior tracking) [30,31,34,36,38,40]. One (6%) intervention used both methods [39]. Of the 11 interventions using sessions [27,28,32,33,35,37,41-45], only 1 (6%) required a session frequency of more than once per week [35]. The interventions using interactions encouraged more regular usage when compared to the interventions involving sessions.

### Comparison

Most of the studies used 2-arm RCT designs [30-34,36,37,39-44] (refer Table 1 for details on comparison groups). A total of 3 RCTs (18%) were classified as 3-arm studies [27,28,38,45] and 1 (6%) used 2 separate 2-arm studies [35].

### Outcomes

All studies reported changes in depression symptoms as their primary outcome, except one [45] that reported it as a secondary outcome. The most commonly reported (>6 studies) secondary outcomes included QoL, functioning and disability, BA, and anxiety. The studies reported these outcomes at various timepoints: 2, 4, 6, 8, 10, 12, 20, 24, 36, 48, and 96 weeks. Studies reported the same outcomes but at different timepoints so when pooling the outcomes at various timepoints for our meta-analysis, only timepoints with at least 2 studies reporting that outcome at that particular timepoint were ultimately chosen. This approach aligns with the concept that a valid quantitative synthesis needs at minimum 2 studies assessing the same outcome at a given timepoint [46]. Refer to Multimedia Appendix 6 [32-36,39-44] for further information on the studies included in the various timepoints for all outcomes ultimately used for the meta-analysis, as well as their significance. Across all studies, the mean (SD) outcome follow-up was 28.2 (SD 23.2) weeks.

### Impact of Digital BA Interventions on Outcomes: Meta-Analysis

The outcomes pooled in our meta-analysis included depression symptoms, anxiety, QoL, BA, functioning and disability, and stress. All interventions were compared to the respective control groups.

#### Impact on Depression Symptoms at 2, 3, 6, and 12 Months

Only 10 studies had sufficient data on depression symptoms for a meta-analysis. The studies with sufficient depression data used various measures, such as the Center for Epidemiologic Studies Depression Scale (CES-D) [42-44], Beck Depression Inventory-II (BDI-II) [39,40], Patient Health Questionnaire -9 (PHQ-9) [32,33], Montgomery–Åsberg Depression Rating Scale (MADRS) [37], Patient Health Questionnaire -8 (PHQ-8) [36], and the Quick Inventory of Depressive Symptomatology – Clinician Rated (QIDS-C) [41]. Of these studies, 8 (80%) were iBA [32,37,39-44] (one of which was a hybrid intervention) and the remaining were one electronic messaging [36] and one telehealth intervention [33]. A total of 3 (30%) studies used a single-component digital BA intervention [36,39,40], while the remaining 7 used multicomponent interventions [32,33,37,41-44]. The intervention groups had statistically significant lower scores on follow-up depression surveys at 2-month [41,43] (n=360, *P*<.001, *I*^2^=0%), 3-month [32,37,44] (n=605, *P*=.001, *I*^2^=51%), and 6-month [33,36,39-42,44] (n=1290, *P*=.009, *I*^2^= 29%) follow-up. However, there were no significant differences at 12 months [33,36] (n=548, *P*=.82, *I*^2^=89%; Multimedia Appendix 7 [32-37,39,40,42]).

#### Impact on BA at 6 Months

BA was measured in 3 studies: one (33%) of them used a single-component digital iBA intervention [35] while the other 2 (67%) studies used multicomponent iBA interventions [41,42]. BA was measured using the Behavioral Activation for Depression Scale (BADS) [35,42] and Behavioral Activation for Depression - Short Form (BADS-SF) [41]. The intervention groups had significantly higher scores on BA surveys at 6 months [35,41,42] (n=1663, *P*<.001, *I*^2^=0%).

#### Impact on Anxiety at 3 and 6 Months

Anxiety was measured in 6 studies: 3 (50%) used single-component interventions [34,39,40] and 3 (50%) used multicomponent interventions [32,37,42]. Studies that reported anxiety outcomes used the Beck Anxiety Inventory (BAI) [37,39,40], General Anxiety Disorder-7 Scale (GAD-7) [34], and the Depression Anxiety Stress Scale -21 (DASS-21) [32]. A total of 5 of the 6 (83%) interventions used iBA [32,37,39,40,42] (including one hybrid intervention) and the remaining intervention used electronic messaging (17%) [34]. The intervention groups did not differ significantly on anxiety at either 3-month [32,34,37] (n=781, *P*=.08, *I*^2^=68%) or 6-month [39,40,42] (n=580, *P*=.24, *I*^2^= 44%) follow-up.

#### Impact on QoL at 3 and 6 Months

QoL was assessed in 6 studies: 4 (67%) of these studies used single-component interventions [34,35,39,40] and 2 (33%) used multicomponent interventions [37,44]. The studies that assessed QoL used the Quality of Life Index (QOLI) [37,40], the 12-Item Short Form Survey (SF-12) [44], EQ-5D-3L [35], EQ-5D-5L [34], and the BAI [39]. A total of 5 (87%) of these studies used iBA [35,37,39,40,44] (including one hybrid intervention) and the remaining study (13%) used electronic messaging [34]. The intervention groups had significantly higher scores on QoL modules at 3 months [34,35,37,44] (n=1937, *P*=.002, *I*^2^=22%) and 6 months [35,39,40,44] (n=1484, *P*=.009, *I*^2^= 0%) follow-up.

#### Impact on Functioning and Disability at 6 Months

A total of 2 studies assessed functioning and disability: 1 (50%) used a single-component iBA intervention [35] while the other study (50%) used a multicomponent iBA intervention [42]. The studies that reported Functioning and Disability used the 12-Item Short Form Survey Physical Component Summary and Mental Component Summary (SF1-12 PCS and MCS) [42] and the World Health Organization Disability Assessment Schedule II (WHODAS-II) [35]. Intervention groups did not differ significantly compared to control groups on modules for functioning and disability at 6-month follow-up [35,42] (n=1942, *P*=.88, *I*^2^=90%).

#### Impact on Stress at 3 Months

**Figure 2:**
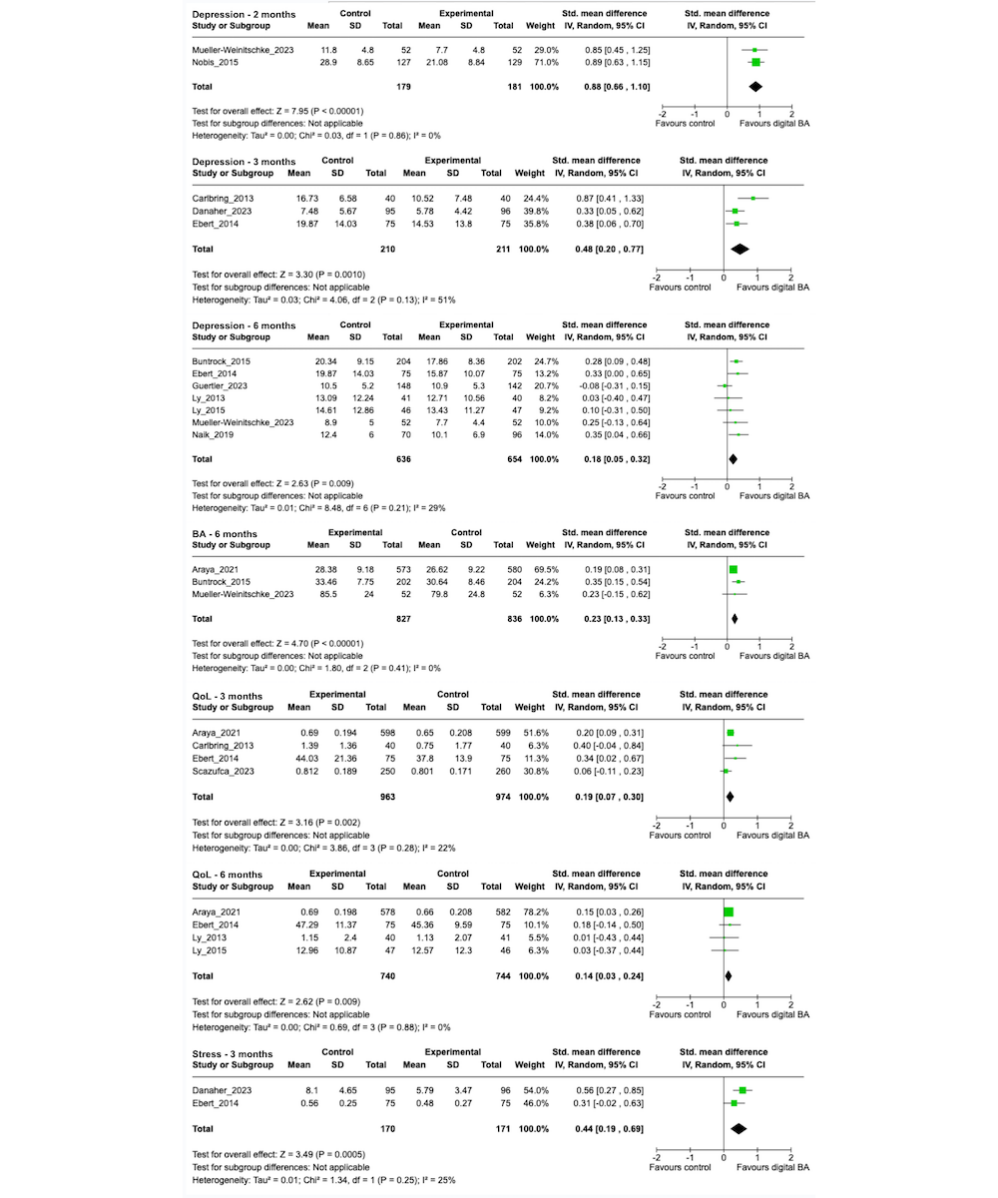
Forest plot of outcomes with statistically significant differences: depression, behavioral activation, quality of life, stress (refer to Multimedia Appendix 7 for forest plots of outcomes that showed no significant differences between intervention and control groups) [32-37,39-44].

A total of 2 studies assessed stress, both of which used multicomponent iBA interventions [32,44]. The studies reported stress using the Perceived Stress Questionnaire (PSQ) [44] and the DASS-21 [32]. The intervention groups scored significantly lower on stress modules at 3 months’ [32,44] (n=341, *P*<.001, *I*^2^=25%) follow-up (Figure 2 [32-37,39-44]).

### Impact of Digital BA Interventions on Outcomes: Narrative Synthesis

The 6 studies that were ineligible for inclusion in the meta-analysis are narratively summarized below.

In addition to BA, Birney et al [31] explored the use of the MoodHacker mobile web app, a self-guided, multicomponent iBA intervention using CBT principles and mindfulness techniques to help working adults with mild-to-moderate depression. The study found significant improvements in depression symptoms, BA, and work-related outcomes at 6 weeks. However, effects were reduced at the 10-week follow-up, suggesting that sustained engagement and support are necessary for long-term efficacy.

Choi et al [27] and its sister study Marti et al [28] assessed the impact of a multicomponent telehealth intervention for depressive symptoms in older adults with comorbid chronic conditions. They compared telehealth CBT, telehealth behavioral activation therapy (BAT), and a TAU control group. Both CBT and BAT significantly improved depressive symptoms and some physical health outcomes up to 3-month follow-up compared to the control. However, CBT showed more substantial long-term reductions in depressive symptoms than BAT. At the 12-month follow-up, neither intervention significantly affected disability, indicating that more nuanced interventions may be necessary for meaningful improvements in physical health.

Jelinek et al [38] evaluated the effects of a brief web-based BA module compared to mindfulness and TAU for individuals with mild depression. While the intervention led to an increase in activity and reduced dysfunctional attitudes, it did not result in a significant reduction in depressive symptoms during the 4-week follow-up.

Sanabria-Mazo et al [45] compared videoconference-delivered ACT and BAT for depression in patients with chronic low back pain and comorbid depressive symptoms. Both interventions produced improvements in depressive symptoms, but neither had a significant impact on functional disability.

Dahne et al [30] evaluated a mobile app–based BA intervention, “Goal2Quit,” combined with nicotine replacement therapy for individuals with depressive symptoms who smoked. The intervention led to reductions in depressive symptoms and increased smoking cessation rates compared to standard self-help materials. Improvements in depression were most notable within the first 3 weeks, and smoking abstinence was significantly higher at weeks 4, 8, and 12.

The narrative synthesis of the 6 studies showed that 5 (87%) of these multicomponent BA interventions produced better results in short-term improvement of depressive symptoms [27,28,30,31,45]. However, these results were not sustained long-term, indicating a need for longer dosing periods. Interventions with outcomes related to physical health [27,28,45] did not produce significant results, suggesting that more tailored interventions may be necessary to improve this aspect.

### RoB of Included Studies

**Figure 3:**
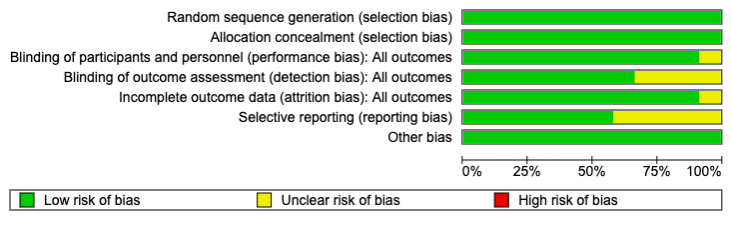
Risk-of-bias summary.

The RoB (Figure 3) was noted as unclear across some studies in the blinding of outcome assessment because these articles did not specify if blinding of the outcome assessments was performed. Refer to Multimedia Appendix 8 [27,30-45] for the full RoB matrix used to generate the summary in Figure 3.

However, all studies were judged to have a low RoB for allocation concealment and random sequence generation.

## Discussion

### Overview

There has been an increasing growth and accessibility of technology in tandem with the rise of mental health threats. This systematic review aimed to aggregate and evaluate empirical evidence on the effectiveness of digital BA interventions designed to alleviate symptoms of either depression or anxiety or both. A total of 18 articles involving 17 RCTs met our inclusion criteria, with 12 (71%) included in the meta-analysis.

### Principal Findings

Most of these studies were conducted in Europe, perhaps because these countries have digital mental health interventions well integrated into their national health systems, with Germany a prime example [47]. The remaining studies were conducted in North and South America. iBA-based interventions were the most common type assessed in the review, potentially due to their relatively low cost, easy access, and general feasibility [48]. Most interventions studied were single-component interventions; the most common multicomponent interventions used BA in addition to problem-solving therapy.

Our meta-analysis found that digital BA interventions significantly reduced depression symptoms at 2, 3, and 6 months, increased BA at 6 months, improved QoL at 3 and 6 months, and relieved stress at 3 months. Only timepoints with at least 2 contributing studies were included in each analysis.

For the significant depression outcomes measured at 2, 3, and 6 months, most interventions were multicomponent, combining BA with other therapeutic elements, which may have contributed to their effectiveness. In particular, many interventions used iBA, which has been shown to be effective at reducing depressive symptoms in prior review articles [16,49].

The BA outcome was found to have a significant effect at 6 months. A total of 2 interventions [41,42] incorporated web-based feedback delivered to patients after each session and optional scheduled text messages to summarize the session discussions. The third study [35] prompted nurse assistants to call participants who were not adhering to the intervention, as well as required mandatory calls at the start.

QoL was found to have significant improvement at 3 and 6 months. For QoL at 3 months, only one of the 4 studies used an intervention that was not targeted for a specific population [37]. Of the 3 studies that addressed specific populations, one study targeted a population with comorbid diabetes or hypertension [35], both of which can negatively alter QoL [50]. Another study targeted older adults in resource-limited situations [34], which also has been shown to be linked with decreased QoL [51]. The last study targeted teachers [44], which as an occupation has been shown to have correlations with decreased QoL [52]. By focusing on the specific challenges affecting each group, these tailored interventions were positioned to yield greater QoL improvements than interventions designed for broader populations. This finding also holds with QoL at 6 months, which also had a significant effect. Of the 4 studies at this timepoint, only the 2 studies which targeted specific populations were found to have significant effects [35,44].

A significant stress reduction was observed at 3 months. The 2 studies that assessed stress at this timepoint also targeted specific populations: perinatal women [32] and teachers [44]. Given that these interventions were tailored to specific populations, they could have been more focused on addressing relevant issues to help alleviate stress, similar to the rationale provided for QoL improvements provided above.

However, the lack of significant effects on depression symptoms at 12 months suggests that benefits may diminish once active treatment concludes. Research on digital therapeutics has identified factors such as reduced motivation, user fatigue, and the loss of novelty in using the app as common obstacles to long-term adherence and effectiveness [53]. Thus, the need for strategies that sustain engagement and continue to reinforce treatment effects beyond the active intervention period is necessary for greater long-term success.

In addition, there were no significant effects found for anxiety at 3 and 6 months. However, given that all interventions were primarily designed to address depression symptoms, these interventions may have been limited in antianxiety components needed to address symptoms like excessive worry or avoidance [54]. Similarly, there was no significant effect on functioning and disability at 6 months. Given that both interventions [35,42] were targeted to addressing mental health rather than physical health, their impact on broader physical functioning may have been limited [55].

The 6 studies included in the narrative synthesis collectively suggest that while digital interventions can be effective in reducing depressive symptoms in the short term, they often fail to produce longitudinal improvements in depression symptoms or anxiety, reinforcing the findings of the meta-analysis [38]. Thus, our meta-analysis and narrative synthesis results indicate that digital BA interventions significantly improve depressive symptoms in the short-term, posttreatment period. Given that most interventions included in our meta-analysis measuring depression outcomes across all timepoints were multicomponent digital BA interventions, we cannot discount the possibility that bundling multiple therapeutic components could have contributed to the significant short-term improvements observed. By addressing a broader range of depression-related factors, these multicomponent formats may reinforce BA’s core mechanisms and enhance overall treatment effectiveness. For these reasons, we recommend considering multicomponent interventions over single-component options in practice.

In addition, it may be beneficial to tailor the intervention to better address issues pertaining to a specific population, such as individuals with a chronic condition. For example, an intervention targeting individuals with diabetes incorporated diabetes-specific themes as an essential part of each session, dedicating significant time in each session to the link between diabetes and depression [35]. This intervention was found to significantly improve depression symptoms up to 2 months posttreatment. In addition, 95% of participants noted that they would recommend the treatment to a friend with diabetes in need of psychological help. Thus, by integrating population-specific themes such as disease education, digital BA can increase relevance and engagement, improving adherence and maximizing therapeutic impact to reduce depression symptoms and anxiety.

### Comparison to Prior Work

This review expands upon previous systematic reviews [20,21] by considering digital BA interventions that use iBA as well as other delivery methods, such as messaging-based interventions and telehealth. The results from this review demonstrate promise for digital BA-based interventions to reduce depressive symptoms, which is consistent with the previous reviews. More specifically, the results from our meta-analysis suggest that multicomponent BA interventions may be more effective at reducing depression symptoms.

### Strengths and Limitations

Our systematic review and meta-analysis are in accordance with PRISMA (Preferred Reporting Items for Systematic Reviews and Meta-Analyses) guidelines (Multimedia Appendix 9), incorporating independent ratings, sensitivity analyses, and an assessment of publication bias. The search strategy was specifically designed to capture a wide range of digital BA interventions, enhancing the thoroughness of our study. However, our approach is not without its limitations. First, the specificity of our search terms, which were tailored to identify interventions explicitly labeled as “behavioral activation,” may have resulted in the exclusion of relevant studies that used different terminology. Despite this, the fact that we captured all studies in our validation set supports the robustness of our search approach. Second, we did not include unpublished research and restricted our search to studies published in English, potentially introducing a language and publication bias. Third, while our meta-analysis provided a detailed examination of the impact of digital BA interventions across various outcomes, the reliance on self-reported measures in most included studies raises concerns about the validity of these findings, particularly in the absence of external assessments of depressive symptoms. Fourth, interventions varied significantly in terms of their format, duration, and intensity, contributing to the observed heterogeneity in our analyses. This variability may have affected the comparability of results across studies. Fifth, the heterogeneity was reduced when interventions were categorized based on the level of guidance provided, suggesting that future meta-analyses might benefit from separating analyses by guidance level to achieve more consistent results. Sixth, the heterogeneity of assessment timepoints across the included studies limited the number of studies included in each meta-analysis, which may have impacted the statistical power and generalizability of our findings. Future research would benefit from more standardized assessment timepoints to enhance comparability and provide more robust conclusions. Seventh, given the rapid pace of change in this field, additional RCTs meeting our eligibility criteria may have been published since our initial review in November 2023. Finally, the different follow-up periods across studies limited our ability to draw conclusions about the long-term efficacy of digital BA interventions. Grouping studies by postmeasurement timepoints in future research could help provide more insight into the stability of intervention effects over time. Nevertheless, this meta-analysis contributes valuable evidence regarding the potential of digital BA interventions to reduce depressive symptoms and enhance mental health outcomes. These findings highlight the potential of digital BA as a scalable and accessible treatment option, offering practical implications for clinical practice and future research.

### Future Directions for Research

We have identified research gaps and opportunities for future work. First, the efficacy of these interventions at least 1 year after completion is difficult to discern. Long-term follow-up studies have shown that while some benefits of digital interventions for depression persist over time, the effects can diminish, and additional interventions might be necessary to maintain mental health improvements [56,57]. Second, the relative efficacies of single-component versus multicomponent digital BA interventions remain unclear. Although this meta-analysis found that multicomponent interventions, especially those combining BA with approaches like problem-solving therapy, often led to greater reductions in depressive symptoms, further research is needed to clarify BA’s specific contribution. Studies involving direct comparisons between single- and multicomponent digital BA interventions could elucidate which approach achieves more consistent and significant benefits across populations [58]. Third, more research is needed to determine whether tailoring interventions, such as through cultural adaptations, improves effectiveness for specific patient populations. Some studies included in this meta-analysis examined populations with chronic pain, perinatal depression, or diabetes. Recent evidence suggests culturally adapted digital interventions are more effective for racial and ethnic minorities than nonadapted controls, suggesting the need to evaluate whether culturally adapted digital BA interventions more effectively reduce depression and anxiety in these populations [59]. Fourth, exploring artificial intelligence–based digital BA interventions may be valuable given the emerging utility of such advanced technologies. Recent studies suggest artificial intelligence can enhance personalization by tailoring treatments to individual patient data. This could improve patient engagement and outcomes by enabling real-time, adaptive adjustments to treatment plans, offering a more responsive approach to managing depression [60]. Fifth, more research assessing the clinical effectiveness of digital BA interventions in other regions, such as Africa, Australia, and Asia, and more studies in South America are needed. Most studies in this review were conducted in Europe, where digital mental health tools have been more readily integrated into health care systems, such as in Germany [47,61]. This integration allows for routine use and evaluation of digital interventions, supported by policies that facilitate data sharing and interoperability with existing care pathways. In contrast, the United States has faced slower adoption potentially due to the fragmented health care system and fewer centralized policies promoting digital mental health [62]. However, progress is being made in the United States, particularly with the introduction of US Food and Drug Administration (FDA) regulations for digital therapeutics, which are paving the way for more standardized and accessible digitally delivered treatments [63]. Addressing these barriers and conducting more studies based in other countries within South America, Asia, and Africa could help establish the effectiveness and scalability of digital BA interventions in global health care contexts. Sixth, the included studies have methodological limitations. Standardized surveys such as the PHQ-9 and BADS-SF provide a simple and standard approach across all patient categories, but this standardization means they may miss patient-specific nuances in depression, BA engagement, or QoL. Patients may also need more time to incorporate these interventions into daily life for full effectiveness. Thus, alternative, individualized assessment methods that are anchored to personal baseline functioning could produce more accurate insights into intervention impact at reducing depression symptoms.

Despite these limitations, digital BA interventions show promise in reducing depression symptoms and anxiety, increasing BA and QoL, and reducing stress. This meta-analysis provides an updated understanding of their utility and efficacy, but ongoing research is crucial as new studies and interventions continuously emerge. In summary, digital BA interventions represent a promising advancement in depression and anxiety treatment, offering accessible, scalable, and potentially effective options for individuals in diverse settings.

### Conclusion

This systematic review and meta-analysis of digital BA interventions found a significant decrease in depression at 2, 3, and 6 months post intervention completion but no significant decrease in depression at 12 months. In addition, the meta-analysis found no significant decrease in anxiety at 3 or 6 months. However, the variability in the significance of depression outcome reductions at different postintervention timepoints suggests a need for further research to determine the long-term efficacy and optimal structuring of these interventions. In addition, the optimal levels of intervention dosage remain unclear, as variations in length and frequency may influence digital BA intervention effectiveness in different ways. The studies included in our meta-analysis used multiple different combinations of frequency and duration, making it difficult to ascertain which regimens were most effective. Future work should focus on understanding the long-term efficacy of digital BA interventions and the effect of intervention dosage (both intervention length and frequency) on efficacy, as well on clarifying the differences between single-component and multicomponent digital BA intervention approaches and cultural adaptability of these interventions to improve depression symptoms and anxiety.

## Appendix

Multimedia Appendix 1 Search strategies.

Multimedia Appendix 2 Inclusion criteria (PICO).

Multimedia Appendix 3 Excluded studies.

Multimedia Appendix 4 Data extraction template.

Multimedia Appendix 5 Definition of terms.

Multimedia Appendix 6 Meta-Analysis Outcomes Summary.

Multimedia Appendix 7 Forest plots of remaining insignificant meta-analyses outcomes [32-37,39,40,42].

Multimedia Appendix 8 Risk of Bias Matrix.

Multimedia Appendix 9 PRSIMA checklist.

## Abbreviations

ACT: acceptance and commitment therapy
BA: behavioral activation
BAI: Beck Anxiety Inventory
BADS: Behavioral Activation for Depression Scale
BADS-SF: Behavioral Activation for Depression - Short Form
BAT: behavioral activation therapy
BDI-II: Beck Depression Inventory-II
CBT: cognitive behavioral therapy
CES-D: Center for Epidemiologic Studies Depression Scale
DASS-21: Depression Anxiety Stress Scale - 21
FDA: US Food and Drug Administration
GAD-7: General Anxiety Disorder-7 Scale
HAPA: Health Action Process Approach
iBA: internet-based BA
MADRS: Montgomery–Åsberg Depression Rating Scale
MCS: Mental Component Summary
PHQ-8: Patient Health Questionnaire-8
PHQ-9: Patient Health Questionnaire-9
PICO: Population, Intervention, Comparison group, and Outcomes
PRISMA: Preferred Reporting Items for Systematic Reviews and Meta-Analyses
PSQ: Perceived Stress Questionnaire
QIDS-C: Quick Inventory of Depressive Symptomatology – Clinician Rated
QoL: quality of life
QOLI: Quality of Life Index
RCT: randomized controlled trial
RoB: risk of bias
SF-12: 12-Item Short Form Survey
SF1-12 PCS: 12-Item Short Form Survey Physical Component Summary
TAU: treatment as usual
WHODAS-II: World Health Organization Disability Assessment Schedule II
