# Supplementary material for: Effectiveness of Digital Behavioral Activation Interventions for Depression and Anxiety: Systematic Review and Meta-Analysis
Source: J Med Internet Res. 2025 Jun 17;27:e68054. doi: 10.2196/68054 (PMC12227033; doi:10.2196/68054)
Supplement: Multimedia Appendix 1 [file jmir_v27i1e68054_app1.docx]

Total: 735
Duplicates: 263
Unique: 472

Embase: 173
Ovid Medline: 179
Web of Science: 128
APA PsycInfo: 94
PubMed excluding Medline: 20
Clinicaltrials.gov: 141

**Embase.com**
173 results on 11/15/23

1. ‘internet’/exp OR ‘computer assisted therapy’/exp OR ‘online system’/exp OR ‘mobile application’/exp OR ‘mobile health’/exp OR ‘smartphone’/exp OR ‘tablet computer’/exp OR ‘video game’/exp OR ‘virtual reality’/exp OR ‘social media’/exp OR ‘telemedicine’/de OR ‘telepsychiatry’/exp OR ‘teletherapy’/exp OR ‘telerehabilitation’/exp OR chat-bot*:ti,ab,kw OR chatbot:ti,ab,kw,de OR webinar*:ti,ab,kw,de OR video-game*:ti,ab,kw OR virtual-reality:ti,ab,kw,de OR social-media:ti,ab,kw,de OR internet:ti,ab,kw,de OR

((online OR digital OR web-based OR website OR internet OR computer* OR multi-media) near/6 (intervention* OR therap* OR treatment* OR rehabilitat* OR program*)):ti,ab,kw,de OR

(mobile near/2 health):ti,ab,kw,de OR mHealth:ti,ab,kw,de OR m-health:ti,ab,kw OR telehealth:ti,ab,kw,de OR tele-health:ti,ab,kw OR telepsychiatry:ti,ab,kw,de OR telerehabilitation:ti,ab,kw,de OR (rehabilitat* near/1 (virtual OR tele)):ti,ab,kw,de OR eHealth:ti,ab,kw,de OR e-health:ti,ab,kw,de OR smartphone*:ti,ab,kw,de OR smart-phone*:ti,ab,kw,de OR (app* near/2 (tablet OR ipad OR mobile OR cell-phone OR phone)):ti,ab,kw,de OR iPhone:ti,ab,kw,de OR iPad:ti,ab,kw,de OR Android-device*:ti,ab,kw,de OR microsoft-surface:ti,ab,kw OR (cell* near/2 phone*):ti,ab,kw,de OR ((computer*) near/2 (Intervention OR remediation OR therap* OR treatment)):ti,ab,kw,de

2. ‘depression’/exp OR ‘anxiety’/exp OR anxiety:ti,ab,kw,de OR anxious:ti,ab OR depression:ti,ab,kw OR depressed:ti,ab,kw OR depressive:ti,ab,kw OR seasonal-affective-disorder*:ti,ab,kw,de OR bipolar-disorder:ti,ab,kw,de OR dysphoria:ti,ab,kw,de OR dysthmia:ti,ab,kw,de OR melancholia:ti,ab,kw,de

3. (‘controlled clinical trial’/exp OR random*:ti,ab,kw OR ‘randomization’/de OR ‘intermethod comparison’/de OR placebo:ti,ab,kw OR (compare or compared or comparison):ti OR ((evaluated or evaluate or evaluating or assessed or assess) and (compare or compared or comparing or comparison)):ab OR (open near/8 label):ti,ab,kw OR ((double or single or doubly or singly) near/8 (blind or blinded or blindly)):ti,ab,kw OR ‘double blind procedure’/de OR parallel-group*:ti,ab,kw OR (crossover or cross over):ti,ab,kw OR ((assign* or match or matched or allocation) near/8 (alternate or group* or intervention* or patient* or subject* or participant*)):ti,ab,kw OR (assigned or allocated):ti,ab,kw OR (controlled near/8 (study or design or trial)):ti,ab,kw OR (volunteer or volunteers):ti,ab,kw OR ‘human experiment’/exp OR trial:ti)

4. ‘behavioral activation’/exp OR (activat* near/5 (behavioural OR behavior*)):ti,ab,kw,de OR (monitor* near/1 (social-activit* OR daily-activit*)):ti,ab,kw,de OR (Self-monitor* near/1 (objective OR subjective)):ti,ab,kw,de OR (mood near/3 activit* near/3 monitor*):ti,ab,kw,de

5. #1 AND #2 AND #3 AND #4

6. #5 AND [english]/lim AND ([article]/lim OR [article in press]/lim OR [data papers]/lim OR [editorial]/lim OR [erratum]/lim OR [letter]/lim OR [note]/lim OR [review]/lim OR [short survey]/lim OR [preprint]/lim)

**OVID MEDLINE**
179 results on 11/15/23

Ovid MEDLINE(R) ALL <1946 to November 14, 2023>

1 exp computer-assisted therapy/ or exp internet/ or exp user-computer interface/ or exp computer-assisted instruction/ or exp Mobile Applications/ or exp handheld computers/ or exp video games/ or exp virtual reality/ or exp social media/ or Telemedicine/ or exp telerehabilitation/ or chat-bot*.ti,ab. or chatbot.mp. or webinar*.mp. or video-game*.ti,ab. or virtual-reality.mp. or social-media.mp. or internet.mp. or ((online or digital or web-based or computer-assist* or website or internet) adj4 (intervention* or therap* or treatment* or rehabilitat*)).mp. or (mobile adj2 health).mp. or mHealth.mp. or m-health.ti,ab. or telehealth.mp. or tele-health.ti,ab. or telepsychiatry.mp. or telerehabilitation.mp. or ((virtual or tele) adj1 (rehabilitat* or guided-mindfulness)).mp. or eHealth.mp. or e-health.mp. or smartphone*.mp. or smart-phone*.mp. or (app* adj2 (tablet or ipad or mobile or cell-phone or phone)).mp. or iPhone.mp. or iPad.mp. or Android-device*.mp. or microsoft-surface.ti,ab. or (cell* adj2 phone*).mp. or (computer* adj2 (Intervention or remediation or therap* or treatment)).mp. or ((online or digital or web-based or website or internet or computer* or multi-media) adj3 (cognitive-behaviour*-therapy or CBT or cognitive-behavior*-therapy or cognitive-behaviour*-intervention or cognitive-behavior*-intervention or cognitive-behaviour*-program* or cognitive-behavior*-program*)).ti,ab. 373520

2 exp depression/ or exp anxiety/ or anxiety.mp. or depression.ti,ab. or depressed.ti,ab. or depressive.ti,ab. or seasonal-affective-disorder*.mp. or bipolar-disorder.mp. or dysphoria.mp. or dysthmia.mp. or melancholia.mp. or anxious.mp. 777021

3 exp randomized controlled trial/ or controlled clinical trial.pt. or randomised.ti,ab. or randomized.ti,ab. or placebo.ab. or randomly.ab. or trial.ab. or groups.ab. 3763677

4 (activat* adj5 (behaviour* or behavior*)).mp. OR (Activit* adj1 (list OR scheduling)).mp. OR

(monitor* adj1 (social-activit* OR daily-activit*)).mp. OR (Self-monitor* adj1 (objective OR subjective)).mp. OR (mood adj3 activit* adj3 monitor*).mp. 11147

5 1 and 2 and 3 and 4 180

6 limit 5 to english language 179

Web of Science
128 results on 11/15/23

Social Sciences Citation Index
Science Citation Index Expanded
Emerging Sources Citation Index

1. (TS= (chat-bot* OR chatbot OR webinar* OR video-game* OR virtual-reality OR social-media OR ((online OR digital OR web-based OR computer-assist* OR website OR internet OR multi-media) near/4 (intervention* OR therap* OR treatment* OR rehabilitat*)) OR (guided-mindfulness near/4 (virtual OR tele)) OR ((app OR apps OR application*) near/2 (tablet OR ipad OR mobile OR cell-phone OR phone)) OR (computer*) near/2 (Intervention OR remediation OR therap* OR treatment)))

2. (TS= (anxiety OR depression OR depressed OR depressive OR seasonal-affective-disorder* OR bipolar-disorder OR dysphoria OR dysthmia OR melancholia ))

3. TI=(random* OR compare or compared or comparison OR trial) OR AB=random* OR KP=(randomization OR ‘intermethod comparison’ OR placebo OR ‘double blind procedure’) OR AB=((evaluated or evaluate or evaluating or assessed or assess) near/30 (compare or compared or comparing or comparison)) OR TS=(open near/8 label) OR TS=((double or single or doubly or singly) near/8 (blind or blinded or blindly)) OR TS=parallel-group* OR TS=(crossover or cross-over OR volunteer OR volunteers) OR TS=((assign* or match or matched or allocation) near/8 (alternate or group* or intervention* or patient* or subject* or participant*)) OR TS=(assigned or allocated) OR TS=(controlled near/8 (study or design or trial))

4. ( TS= (activat* near/5 (behavioural OR behavior*)) OR (monitor* near/1 (social-activit* OR daily-activit*)) OR (Self-monitor* near/1 (objective OR subjective)) OR (mood near/3 activit* near/3 monitor*))

5. #1 AND #2 AND #3 AND #4

6. #5 AND Article or Review Article or Early Access or Letter (Document Types) AND English (Languages)

APA PsycInfo
94 results on 11/15/23

1. TX (depression OR anxiety)
2. TX (online OR digital OR web-based OR computer-assist* OR website OR internet OR multi-media OR chat-bot* OR chatbot OR webinar* OR video-game* OR virtual-reality OR social-media OR virtual OR app OR apps OR application OR tablet OR ipad OR mobile OR cell-phone OR phone OR (computer N2 (Intervention OR remediation OR therap* OR treatment)) )

3. DE “Behavioral Activation System” OR TX (“behavioral activation” OR “behavioural activation”) OR TX (Activit* N1 (list OR scheduling)) OR TX (monitor* N1 (social-activit* OR daily-activit*)) OR TX (Self-monitor* N1 (objective OR subjective))

4. 1 AND 2 AND 3

5. Narrow by Methodology: - clinical trial

PubMed
20 results on 11/15/23

(randomized[title] OR randomised[title] OR trial[title]) AND (("online"[All Fields] OR ("digital"[All Fields] OR "digitalisation"[All Fields] OR "digitalised"[All Fields] OR "digitalization"[All Fields] OR "digitalize"[All Fields] OR "digitalized"[All Fields] OR "digitalizer"[All Fields] OR "digitalizing"[All Fields] OR "digitally"[All Fields] OR "digitals"[All Fields] OR "digitization"[All Fields] OR "digitizations"[All Fields] OR "digitize"[All Fields] OR "digitized"[All Fields] OR "digitizer"[All Fields] OR "digitizers"[All Fields] OR "digitizes"[All Fields] OR "digitizing"[All Fields]) OR "web-based"[All Fields] OR "computer assist*"[All Fields] OR ("website"[All Fields] OR "website s"[All Fields] OR "websites"[All Fields]) OR ("internet"[MeSH Terms] OR "internet"[All Fields] OR "internet s"[All Fields] OR "internets"[All Fields])) AND ("depressed"[All Fields] OR "depression"[MeSH Terms] OR "depression"[All Fields] OR "depressions"[All Fields] OR "depression s"[All Fields] OR "depressive disorder"[MeSH Terms] OR ("depressive"[All Fields] AND "disorder"[All Fields]) OR "depressive disorder"[All Fields] OR "depressivity"[All Fields] OR "depressive"[All Fields] OR "depressively"[All Fields] OR "depressiveness"[All Fields] OR "depressives"[All Fields] OR ("anxiety"[MeSH Terms] OR "anxiety"[All Fields] OR "anxieties"[All Fields] OR "anxiety s"[All Fields])) AND ("behavioral-activation"[All Fields] OR "behavioural-activation"[All Fields])) NOT (medline[Filter])

**Clinicaltrials.gov**
141 results on 11/15/23

Condition or disease: depression OR anxiety
Other Terms: digital OR multi-media OR web-based OR online OR internet OR computer-based OR computer-assisted
Intervention: “behavioural activation” OR “Behavioral Activation”
