# Supplementary material for: Effectiveness of Digital Behavioral Activation Interventions for Depression and Anxiety: Systematic Review and Meta-Analysis
Source: J Med Internet Res. 2025 Jun 17;27:e68054. doi: 10.2196/68054 (PMC12227033; doi:10.2196/68054)
Supplement: Multimedia Appendix 2 [file jmir_v27i1e68054_app2.docx]

• P: Adults at least 18 years old who screened positive for symptoms of anxiety and /or depression

• I: Digital mental health interventions utilizing BA. This may include but not limited to:

smartphone and tablet applications, internet based programs, virtual reality, media based

programs, video game consoles, computer programs, chat bots, telehealth, social media,

podcasts, and webinars.

• C: Study comparator: RCT

• O: Anxiety and / or depression
