# Supplementary material for: Effectiveness of Digital Behavioral Activation Interventions for Depression and Anxiety: Systematic Review and Meta-Analysis
Source: J Med Internet Res. 2025 Jun 17;27:e68054. doi: 10.2196/68054 (PMC12227033; doi:10.2196/68054)
Supplement: Multimedia Appendix 3 [file jmir_v27i1e68054_app3.docx]

|  | **Author** | **Year** | **Reason for exclusion** |
| --- | --- | --- | --- |
| 1 | Akechi | 2022 | Population did not require prior anxiety and / or depression |
| 2 | Arjadi | 2016 | Study protocol |
| 3 | Bombardier | 2009 | Study population not strictly adults |
| 4 | Choi | 2020 | Population did not require prior anxiety and / or depression |
| 5 | Dahne (Aptivate…) | 2019 | Feasibility study |
| 6 | Dahne (Self Help Behavioral…) | 2019 | Feasibility study |
| 7 | Deady | 2018 | Study protocol |
| 8 | Deady | 2020 | Population did not require prior anxiety and / or depression |
| 9 | Drew | 2021 | Retrospective study |
| 10 | Economides | 2022 | Feasibility study |
| 11 | Egede | 2009 | Study protocol |
| 12 | Egede | 2015 | Study protocol |
| 13 | Eisma | 2015 | Feasibility study |
| 14 | Fu | 2021 | Study population not strictly adults |
| 15 | Gomez-Cambronero | 2023 | Study protocol |
| 16 | Grenawalt | 2022 | Study population not strictly adults |
| 17 | Hart | 2019 | Outcome not related to anxiety and / or depression |
| 18 | Imai | 2020 | Retrospective study |
| 19 | Lambert | 2018 | Feasibility study |
| 20 | Lewis | 2017 | Population did not require prior anxiety and / or depression |
| 21 | Lüdtke | 2018 | Not a RCT |
| 22 | Luxton | 2014 | Study protocol |
| 23 | Ly | 2012 | Study protocol |
| 24 | MacPherson | 2010 | Intervention is not digital |
| 25 | Meyer | 2009 | Population did not require prior anxiety and / or depression |
| 26 | O'Mahen | 2013 | Feasibility study |
| 27 | O’Mahen | 2014 | Feasibility study |
| 28 | Paul | 2022 | Feasibility study |
| 29 | Pruitt | 2018 | Outcome not related to anxiety and / or depression |
| 30 | Puspitasari | 2017 | Population did not require prior anxiety and / or depression |
| 31 | Reitsma | 2023 | Intervention not BA |
| 32 | Renner | 2016 | Outcome not related to anxiety and / or depression |
| 33 | Ruzickova | 2021 | Study population not strictly adults |
| 34 | Taylor | 2023 | Feasibility study |
| 35 | Trombello | 2020 | Not a RCT |
| 36 | Turner | 2019 | Population did not require prior anxiety and / or depression |
