# Supplementary material for: Effectiveness of Digital Behavioral Activation Interventions for Depression and Anxiety: Systematic Review and Meta-Analysis
Source: J Med Internet Res. 2025 Jun 17;27:e68054. doi: 10.2196/68054 (PMC12227033; doi:10.2196/68054)
Supplement: Multimedia Appendix 4 [file jmir_v27i1e68054_app4.docx]

| Study ID |  |
| --- | --- |
| Location |  |
| Sample Size - treatment |  |
| Sample Size - control |  |
| Percent Female - treatment |  |
| Percent Male - treatment |  |
| Percent Female - control |  |
| Percent Male - control |  |
| Recruitment Strategy |  |
| Arms |  |
| Study Design |  |
| Outcome Reporting |  |
| Anxiety / Depression |  |
| Inclusion criteria |  |
| Exclusion criteria |  |
| Intervention Name |  |
| Intervention Components |  |
| Number of components |  |
| Mechanisms of change |  |
| Content Type |  |
| Intervention length |  |
| Guidance |  |
| Assistance |  |
| Assistance Type |  |
| Intervention Targets |  |
| Curriculum Type |  |
| Follow up duration |  |
| Technology type |  |
| Control Type |  |
| Primary Outcomes |  |
| Primary Outcome results |  |
| Secondary Outcomes |  |
| Secondary outcome results |  |
| Lost to follow up |  |
