# Supplementary material for: Effectiveness of Digital Behavioral Activation Interventions for Depression and Anxiety: Systematic Review and Meta-Analysis
Source: J Med Internet Res. 2025 Jun 17;27:e68054. doi: 10.2196/68054 (PMC12227033; doi:10.2196/68054)
Supplement: Multimedia Appendix 5 [file jmir_v27i1e68054_app5.docx]

| **Term** | **Definition** |
| --- | --- |
| iBA | A web-based intervention that helps patients with depression by developing new behavioral activities |
| Electronic messaging | Interventions that deliver periodic prompts to promote behavioral change |
| Telehealth | Interventions aiming to provide healthcare services remotely using electronic devices |
| Self-paced | Interventions where the user determines how fast they progress through the intervention |
| Guided | Interventions where the order of modules are predetermined for the user |
| Multi-component | Interventions with another form of psychotherapy, in addition to BA |
| Healthcare | Patients recruited from or referred by hospitals, healthcare systems, or case managers |
