# Supplementary material for: Effectiveness of Digital Behavioral Activation Interventions for Depression and Anxiety: Systematic Review and Meta-Analysis
Source: J Med Internet Res. 2025 Jun 17;27:e68054. doi: 10.2196/68054 (PMC12227033; doi:10.2196/68054)
Supplement: Multimedia Appendix 6 [file jmir_v27i1e68054_app6.docx]

**Table S1.**

| Outcome | Included studies | Significant? |
| --- | --- | --- |
| Depression Symptoms - 2 months | Mueller-Weinitschke et al (2023) [41] , Nobis et al (2015) [43] | Yes |
| Depression Symptoms - 3 months | Carlbring et al (2013) [37], Danaher et al (2023) [32], Ebert et al (2014) [44] | Yes |
| Depression Symptoms - 6 months | Buntrock et al (2015) [42], Ebert et al (2014) [44], Guertler et al (2023) [36], Ly et al (2013) [40], Ly et al (2015) [39], Mueller-Weinitschke et al (2023) [41], Naik et al (2019) [33] | Yes |
| Depression Symptoms - 12 months | Guertler et al (2023) [36], Naik et al (2019) [33] | No |
| Behavioral Activation - 6 months | Araya et al (2021) [35], Buntrock et al (2015) [42], Mueller-Weinitschke et al (2023) [41] | Yes |
| Anxiety - 3 months | Carlbring et al (2013) [37], Danaher et al (2023) [32], Scazufca et al (2024) [34] | No |
| Anxiety - 6 months | Buntrock et al (2015) [42], Ly et al (2013) [40], Ly et al (2015) [39] | No |
| Quality of Life - 3 months | Araya et al (2021) [35], Carlbring et al (2013) [37], Ebert et al (2014) [44], Scazufca et al (2024) [34] | Yes |
| Quality of Life - 6 months | Araya et al (2021) [35], Ebert et al (2014) [44], Ly et al (2013) [40], Ly et al (2015) [39] | Yes |
| Functioning/Disability - 6 months | Araya et al (2021) [35], Buntrock et al (2015) [42] | No |
| Stress - 3 months | Danaher et al (2023) [32], Ebert et al (2014) [44] | Yes |

### References

32. Danaher, B.G., et al., Trial of a patient-directed eHealth program to ameliorate perinatal depression: the MomMoodBooster2 practical effectiveness study. American Journal of Obstetrics and Gynecology, 2023. 228(4): p. 453.e1-453.e10.

33. Naik, A.D., et al., Effect of Telephone-Delivered Collaborative Goal Setting and Behavioral Activation vs Enhanced Usual Care for Depression Among Adults With Uncontrolled Diabetes: A Randomized Clinical Trial. JAMA Network Open, 2019. 2(8): p. e198634.

34. Scazufca, M., et al., Self-help mobile messaging intervention for depression among older adults in resource-limited settings: a randomized controlled trial. Nature Medicine, 2024. 30(4): p. 1127-1133.

35. Araya, R., et al., Effect of a Digital Intervention on Depressive Symptoms in Patients With Comorbid Hypertension or Diabetes in Brazil and Peru: Two Randomized Clinical Trials. JAMA, 2021. 325(18): p. 1852.

36. Guertler, D., et al., E-Health intervention for subthreshold depression: Reach and two-year effects of a randomized controlled trial. Journal of Affective Disorders, 2023. 339: p. 33-42.

39. Ly, K.H., et al., Smartphone-Supported versus Full Behavioural Activation for Depression: A Randomised Controlled Trial. PLOS ONE, 2015. 10(5): p. e0126559.

40. Ly, K.H., et al., Behavioural activation versus mindfulness-based guided self-help treatment administered through a smartphone application: a randomised controlled trial. BMJ Open, 2014. 4(1): p. e003440.

41. Mueller-Weinitschke, C., et al., Effects of a Web-Based Behavioral Activation Intervention on Depressive Symptoms, Activation, Motivation, and Volition: Results of a Randomized Controlled Trial. Psychotherapy and Psychosomatics, 2023. 92(6): p. 367-378.

42. Buntrock, C., et al., Effectiveness of a Web-Based Cognitive Behavioural Intervention for Subthreshold Depression: Pragmatic Randomised Controlled Trial. Psychotherapy and Psychosomatics, 2015. 84(6): p. 348-358.

43. Nobis, S., et al., Efficacy of a Web-Based Intervention With Mobile Phone Support in Treating Depressive Symptoms in Adults With Type 1 and Type 2 Diabetes: A Randomized Controlled Trial. Diabetes Care, 2015. 38(5): p. 776-783.

44. Ebert, D.D., et al., Efficacy of an internet-based problem-solving training for teachers: results of a randomized controlled trial. Scandinavian Journal of Work, Environment & Health, 2014. 40(6): p. 582-596.
