# Supplementary figures and images for: Effectiveness of Digital Behavioral Activation Interventions for Depression and Anxiety: Systematic Review and Meta-Analysis
Source: J Med Internet Res. 2025 Jun 17;27:e68054. doi: 10.2196/68054 (PMC12227033; doi:10.2196/68054)

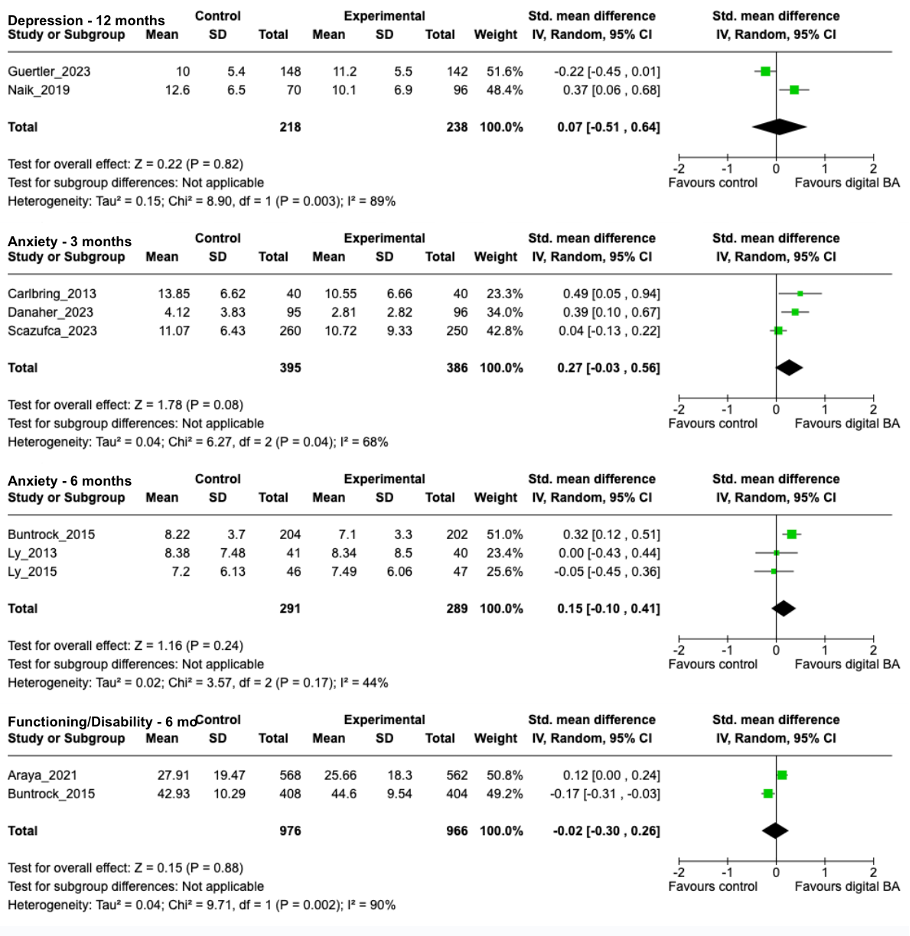

Supplement: Multimedia Appendix 7 [file jmir_v27i1e68054_app7.png]
