# Supplementary material for: Effectiveness of Digital Behavioral Activation Interventions for Depression and Anxiety: Systematic Review and Meta-Analysis
Source: J Med Internet Res. 2025 Jun 17;27:e68054. doi: 10.2196/68054 (PMC12227033; doi:10.2196/68054)
Supplement: Multimedia Appendix 8 [file jmir_v27i1e68054_app8.docx]

**Table S1.**

| Studies | Random sequence generation | | Allocation concealment | | Blinding of participants and personnel | | Blinding of outcome assessment | | Incomplete outcome data | | Selective outcome reporting | | Other bias | |
| --- | --- | --- | --- | --- | --- | --- | --- | --- | --- | --- | --- | --- | --- | --- |
| Reviewer | JM | EJ | JM | EJ | JM | EJ | JM | EJ | JM | EJ | JM | EJ | JM | EJ |
| Araya et al (2021) [35] | Low | Low | Low | Unclear | Low | High | High | High | Low | Low | Unclear/Low | Low | Unclear/Low | Low |
| Birney et al (2016) [31] | Low | Low | Unclear/Low | Unclear | Low | High | High | High | Low | Low | Unclear | Unclear | Low | Low |
| Buntrock et al (2015) [42] | Low | Low | Low | Low | Low | High | High | High | Unclear/Low | Low | Unclear | Unclear | Unclear | Low |
| Carlbring et al (2013) [37] | Low | Low | Low | Low | Low | High | High | Moderate | Low | Low | Unclear | Low | Low | Low |
| Choi et al (2020), [27] | Low | Low | Low | Unclear | Low | High | Medium | Low | Low | Moderate | Unclear/Low | Unclear | Low | Moderate |
| Dahne et al (2023) [30] | Unclear/Low | Low | Low | Unclear | Low | High | High | Unclear | Low | Low | Unclear | Low | Unclear | Low |
| Danaher et al (2023) [32] | Unclear/Low | Low | Low | Unclear | Low | High | High | High | Low | Low | Unclear | Low | Low | Low |
| Ebert et al (2014) [44] | Low | Low | Low | Low | Low | High | High | High | Low | Low | Unclear | Low | Low | Low |
| Guertler et al (2023) [36] | Low | Low | Low | Low | Low | High | High | Moderate | Low | Low | Unclear | Low | Low | Low |
| Jelinek et al (2020) [38] | Unclear/Low | Low | Low | Low | Low | High | High | High | Unclear/Low | Low | Unclear | Low | Low | Low |
| Ly (2013) et al (2013) [40] | Unclear/Low | Low | Low | Low | Low | High | High | High | Low | Low | Unclear | Low | Low | Low |
| Ly (2014) et al (2015) [39] | Low | Low | Low | Low | Low | High | Unclear/High | High | Low | Low | Unclear | Low | Unclear | Low |
| Mueller-Weinitschke et al (2023) [41] | Low | Low | Low | Low | Low | High | High | Unclear/high | Low | Low | Low | Low | Low | Low |
| Naik et al (2019) [33] | Unclear/Low | Low | Low | Low | Low | High | High | High | Low | Low | Low | Low | Low | Low |
| Nobis et al (2015) [43] | Low | Low | Low | Low | Unclear/Low | High | High | High | Unclear/High | Unclear | Unclear | Low | Low | Low |
| Sanabria-Mazo et al (2023) [45] | Low | Low | Low | Low | Low | High | High | High | Unclear/Low | Low | Unclear | Low | Unclear | Low |
| Scazufca et al (2024) [34] | Low | Low | Low | Low | Low | High | High | High | Low | Low | Unclear | Low | Low | Low |

### References

27. Choi, N.G., et al., Effect of Telehealth Treatment by Lay Counselors vs by Clinicians on Depressive Symptoms Among Older Adults Who Are Homebound: A Randomized Clinical Trial. JAMA Network Open, 2020. 3(8): p. e2015648.

30. Dahne, J., et al., Behavioral Activation–Based Digital Smoking Cessation Intervention for Individuals With Depressive Symptoms: Randomized Clinical Trial. Journal of Medical Internet Research, 2023. 25: p. e49809.

31. Birney, A.J., et al., MoodHacker Mobile Web App With Email for Adults to Self-Manage Mild-to-Moderate Depression: Randomized Controlled Trial. JMIR mHealth and uHealth, 2016. 4(1): p. e8.

32. Danaher, B.G., et al., Trial of a patient-directed eHealth program to ameliorate perinatal depression: the MomMoodBooster2 practical effectiveness study. American Journal of Obstetrics and Gynecology, 2023. 228(4): p. 453.e1-453.e10.

33. Naik, A.D., et al., Effect of Telephone-Delivered Collaborative Goal Setting and Behavioral Activation vs Enhanced Usual Care for Depression Among Adults With Uncontrolled Diabetes: A Randomized Clinical Trial. JAMA Network Open, 2019. 2(8): p. e198634.

34. Scazufca, M., et al., Self-help mobile messaging intervention for depression among older adults in resource-limited settings: a randomized controlled trial. Nature Medicine, 2024. 30(4): p. 1127-1133.

35. Araya, R., et al., Effect of a Digital Intervention on Depressive Symptoms in Patients With Comorbid Hypertension or Diabetes in Brazil and Peru: Two Randomized Clinical Trials. JAMA, 2021. 325(18): p. 1852.

36. Guertler, D., et al., E-Health intervention for subthreshold depression: Reach and two-year effects of a randomized controlled trial. Journal of Affective Disorders, 2023. 339: p. 33-42.

37. Carlbring, P., et al., Internet-based behavioral activation and acceptance-based treatment for depression: A randomized controlled trial. Journal of Affective Disorders, 2013. 148(2-3): p. 331-337.

38. Jelinek, L., et al., Brief Web-Based Intervention for Depression: Randomized Controlled Trial on Behavioral Activation. Journal of Medical Internet Research, 2020. 22(3): p. e15312.

39. Ly, K.H., et al., Smartphone-Supported versus Full Behavioural Activation for Depression: A Randomised Controlled Trial. PLOS ONE, 2015. 10(5): p. e0126559.

40. Ly, K.H., et al., Behavioural activation versus mindfulness-based guided self-help treatment administered through a smartphone application: a randomised controlled trial. BMJ Open, 2014. 4(1): p. e003440.

41. Mueller-Weinitschke, C., et al., Effects of a Web-Based Behavioral Activation Intervention on Depressive Symptoms, Activation, Motivation, and Volition: Results of a Randomized Controlled Trial. Psychotherapy and Psychosomatics, 2023. 92(6): p. 367-378.

42. Buntrock, C., et al., Effectiveness of a Web-Based Cognitive Behavioural Intervention for Subthreshold Depression: Pragmatic Randomised Controlled Trial. Psychotherapy and Psychosomatics, 2015. 84(6): p. 348-358.

43. Nobis, S., et al., Efficacy of a Web-Based Intervention With Mobile Phone Support in Treating Depressive Symptoms in Adults With Type 1 and Type 2 Diabetes: A Randomized Controlled Trial. Diabetes Care, 2015. 38(5): p. 776-783.

44. Ebert, D.D., et al., Efficacy of an internet-based problem-solving training for teachers: results of a randomized controlled trial. Scandinavian Journal of Work, Environment & Health, 2014. 40(6): p. 582-596.

45. Sanabria-Mazo, J.P., et al., Efficacy of Videoconference Group Acceptance and Commitment Therapy (ACT) and Behavioral Activation Therapy for Depression (BATD) for Chronic Low Back Pain (CLBP) Plus Comorbid Depressive Symptoms: A Randomized Controlled Trial (IMPACT Study). The Journal of Pain, 2023. 24(8): p. 1522-1540.
